# Reliability and Validity Evidences of Tej Emotional and Behavioral Problem Checklist (TEJ-CL) for Child Mental Health Assessment in Nepal

**DOI:** 10.31729/jnma.8785

**Published:** 2024-10-31

**Authors:** Suraj Shakya, Sabitri Sthapit, Mita Rana, Arun Raj Kunwar, Shital Bhandary

**Affiliations:** 1 Institute of Medicine, Tribhuvan University, Maharajgunj, Kathmandu, Nepal; 2 Central Department of Psychology, Tribhuvan University, Kirtipur, Nepal; 3 Kanti Children's Hospital, Maharajgunj, Kathmandu, Nepal; 4 Patan Academy of Health Sciences, Lagankhel, Lalitpur, Nepal

**Keywords:** *behavior rating scale*, *Nepalese children*, *psychometrics*, *Tej*

## Abstract

**Introduction:**

The Tej Emotional and Behavioral Problem Checklist (TEJ-CL) was developed in Nepalese context to aid assessment of childhood emotional and problems of children. This study aimed to evaluate TEJ-CL's factor structure, reliability, and validity evidences as an add-on and symptom monitoring test.

**Methods:**

This cross-sectional validation study included guardians of 320 children (age 5–17 years) from tertiary mental health centers in Kathmandu as referred group, along with 601 children from two schools (private and community) in Kathmandu as non-referred group. IRC was obtained ethical approval (ref: 183 (6-11-E)2/073/074 and ref: 776). TEJ-CL, an 89-item parent-reported questionnaire, served as the index test, while referral status acted as the reference standard. Factor structure, internal consistency, test-retest/cross-informant correlations and criterion validity evidence was assessed using principal component analysis, coefficient alpha, spearman's rank correlation and linear regression models, respectively.

**Results:**

Analysis was done using 179 referred and 412 non-referred individuals based on non-missing data. Principal component analysis in referred sample reduced the number of items of questionnaire to 65 from 89 and indicated six factors: externalizing behavioral issues, anxiety/worries, upset/sadness, somatic concern, miscellaneous syndrome, and severe issues with coefficient alpha ranging from .62 to .95. As criterion validity evidence, referred children showed significantly higher scores than non-referred children across composite and factor scores, except for anxiety/worries factor. Similarly, regression analyses within the referred group demonstrated significant associations between factor scores and specific diagnoses.

**Conclusions:**

Reliability and validity estimate of questionnaire is comparable to similar empirically based scales. Future research should focus on assessing the tool's generalizability and improving discriminatory indexes.

## INTRODUCTION

Accurate assessment of emotional and behavioral problems in children is crucial for early intervention and support. Still, existing tools in the Nepalese context often encounter challenges of cultural appropriateness, cost to import from international distributors and complexity in use of tool for nonspecialists in children mental health.^1-4^

The Tej Emotional and Behavioral Problem Checklist (TEJ-CL) is parent rating scale in Nepali language developed for the context of Nepal. It primarily serves as an add-on test in clinical setting to improve accuracy of assessment of child psychopathology and as a symptom monitoring test during intervention process.^5^ In addition, it is a potential screening tool in community setting by school nurses and counselors through task shifting,^1^ enabling them to effectively screen childhood psychopathology. It incorporates empirical and dimensional based approach to psychopathology and follows similar trend of tool development.^6-9^

The purpose of this paper is to present overview of TEJ-CL validation procedure, especially evaluating its factor structure, reliability evidence, and concurrent criterion validity evidence^10^ as a potential add-on and symptom monitoring test.

## METHODS

It was a cross-sectional, validation study. The study was part of the first author's doctoral research project. Data were collected during 2017 from TU Teaching Hospital (TUTH), Kanti Children's Hospital (KCH), a private and a community school from Kathmandu. Ethical approval (IRC approval number[ref: 183 (6-11-E)2/073/074) was obtained from the institutional review boards at TUTH and KCH. Informed written consent was obtained from all participating guardians after a detailed explanation of the study procedures. The participants were told that their responses would be utilized for refinement of the questionnaire on emotional and behavioral problems.

Participants for the research were the parents or guardians of children aged 5 to 17 years. The participants for the referred or high-risk group were purposefully selected guardians who brought their children to child mental health facilities at TUTH and KCH. Children suspected of pervasive developmental disorder or intellectual disability by the clinicians were excluded. Similarly, the 'non-referred' or community comparison group is comprised of the guardians purposefully selected from a private and a community school in Kathmandu. A request letter was sent to the whole section of each grade (grade 1 till 10). The sections of each grade were chosen conveniently based on suggestion by the school administration. No other exclusions based on risk of emotional and behavioral problems were done.

Sample size for the referred group was determined based on two criteria, considering a minimum of 150-300 participants for exploratory factor analysis, as well as three times the number of items in questionnaire as shown below.^11^

n = 3×89 = 267

To account for a potential non-response rate of 20%, this number was inflated by 20%.

267×1.2 = 320.4 ~ 320

Hence, we opted for 320 participants in this group and collected data from 320 samples. For community sample, we opted for approximately double the size i.e. 640 for comparison, and collected data from 601 participants.^12^

We have used questionnaires with non-missing data while conducting exploratory factor analysis in order to include responses for all the items to ensure validity and reliabilities of the tool.

Index Test and reference standard: TEJ-CL was an index test, which is an 89-item parent-reported printed questionnaire in Nepali, developed exclusively for use in Nepal. Response options were a 3-point Likert scale, based on parental perception of the problem items being concerning for the last 2 months. It was developed iteratively during the initial phase of the same research through a multi-stage process. The item pool for TEJ-CL was generated from a free listing of chief complaints from 154 referred children at TUTH and KCH during 2014-2016. Feasibility study was conducted among 25 referred and 145 non-referred children in Kathmandu. Modification was made based on cognitive interviews, results of item analysis, expert consultation and adequacy check with child mental health syndromes in the International Classification of Disease (ICD-10) and Diagnostic and Statistical Manual of Mental Disorders (DSM-5). Even though nine items had low indices for discrimination, endorsement, and item total correlation (<0.30), they were retained for their clinical significance (e.g. suicide related item). We checked three-point response scale and four-point response scale in cognitive interview and later decided on three-point response scale. This served two purposes - first it gave opportunity for relatively neutral response for the guardians, and it was easier for those with relatively lesser reading competency. The final draft underwent content validity assessment by the 10 experts in psychiatry and clinical psychology [Content Validity Index (CVI)=0.86] for the overall questionnaire. Though the questionnaire may have potential utility as a screening tool in the community, the current article presents evidence for its utility as add-on tool and symptom monitoring tool in a clinical setting.

The referral status, indicating whether children were referred to a clinic or not, served as the reference standard for the study. The referred population served as at-risk population, or 'caseness' for emotional and behavioural problems. Similarly, non-referred population represented general population, with a mixture of at risk as well as non-risk population.

In the clinical setting, the first author, two volunteer master-level psychology students and four residents of TUTH as data enumerators. They were oriented on research purpose and procedures by the first author. The enumerators briefed the guardians about the study, took verbal consent and provided TEJ-CL and helped in clarification or reading of the items only when required. The enumerators had no role in further analysis of the results of TEJ-CL. In the school setting, the guardians were sent with sealed envelope containing request letter, information of research and TEJ-CL. They were sent via students through their respective class teachers.

As a stringent criterion, forms without any missing values for 89 problem items were used for the analysis, and only 179 referred and 412 non-referred samples fulfilled this criterion. However, we still fulfilled our minimum sample requirement for factor analysis and group comparison. Principal Component analysis (PCA) was conducted for 179 forms in the referred group after confirming suitability criteria like: sample size > 150, inter-item correlations >0.3, and acceptable measures of sampling adequacy.^11^ Determining the number of factors/components involved a threepronged strategy: Kaiser's criterion (Eigenvalue > 1, adjusted to 2 in the final version), Cattell's scree test, and Horn's parallel Analysis comparing Eigenvalues to randomly generated data.^13^ Finally, the factors were rotated using the varimax method for clearer interpretation. Internal consistency of the composite score and sub scales (factors) were assessed using coefficient alpha. Cross informant correlation among the fathers and mothers and test retest reliability in 1-week period were assessed using spearman's rank correlation (rs). Well-known parental reporting measure (Child Behavior Checklist)^14^ has mean rs of 0.75 and 0.80 for cross informant correlation (parental agreement) of syndrome scales and total score. It has mean rs of 0.89 for test retest reliability (within 8-16 days) for syndrome scales, while 0.94 for total score. For criterion validity, the criterion is referral status, and this was assessed for the composite score as well as factor scores controlling for socio demographic variables found to be statistically significant (p<0.05) during the bivariate regression as well as absence of multicollinearity (Variance Inflation Factor <10) among them. Regression equations are useful ways to link test scores with criterion performance.^10^ JASP software version 0.14.315 was used for all the statistical analysis.^15^

## RESULTS

In the analyzed dataset (n=179 referred and n=412 non-referred, excluding missing data), 77 (43.02%) were aged 6-11, while 102 (56.98%) were aged 12-16 in referred group. Similarly, 168 (40.77%) were aged 6-11, and 244 (59.22%) were aged 12-16 in non-referred group. There was a balanced distribution of boys and girls in both groups. Province information was only available for referred children, with the majority 74 (41.34%) residing in Bagmati Province. Mothers were the primary reporters in both referred 84 (46.93%) and non-referred 202 (49.02%) groups, followed by fathers (42.46% in referred and 38.83% in non-referred). In terms of guardian education, a higher proportion of guardian's in non-referred had secondary or higher education 342 (83.01%) compared to 123 (68.72%) in the referred group. A small percentage of guardians in both groups were either literate or had only primary education (27.38% in referred and 12.13% in nonreferred). Referred children had various conditions, including Attention Deficit Hyperactivity Disorder-ADHD 16 (8.94%), anxiety 37 (20.67%), and conduct disorders 12 (17.32%), as well as other psychiatric issues like adjustment disorder, depression, psychosis, and psychosomatic problems. A few children with specific learning disabilities, school refusal, stuttering, borderline traits, and bipolar disorder were grouped under 'other' categories (Table 1).

**Table 1:** Demographic characteristics^*^

Characteristics	Referral Status		
	Referred (n = 179)		Non-referred (n=412)
	n (%)		n (%)
Age	6-11	77 (43.02)	168 (40.77)
	12-16	102 (56.98)	244 (59.22)
Sex	Not mentioned	15 (8.38)	30 (7.28)
	Male	90 (50.28)	179 (43.44)
	Female	74 (41.34)	203 (49.27)
Province (residence)	Not mentioned	8 (4.47)	
	Province 1	6 (3.35)	
	Province 2	5 (2.79)	
	Province 3	74 (41.34)	
	Province 4	12 (6.70)	
	Province 5	52 (29.05)	
	Province 6	11 (6.15)	
	Province 7	11 (6.15)	
Type of schooling	Not mentioned	61 (34.08)	3 (0.72)
	Community	30 (16.76)	53 (12.86)
	Private	88 (49.16)	356 (86.41)
Relationship	Not mentioned	1 (0.56)	4 (0.97)
	Mother	84 (46.93)	202 (49.02)
	Father	76 (42.46)	160 (38.83)
	Relatives	12 (6.70)	33 (8.01)
	Others	6 (3.35)	13 (3.16)
Education of Guardian	Not mentioned	7 (3.91)	20 (4.85)
	Literate	17 (9.50)	10 (2.42)
	Primary	32 (17.88)	40 (9.71)
	Secondary	54 (30.17)	110 (26.70)
	Higher education	69 (38.55)	232 (56.31)
Diagnosis	Not mentioned	33 (18.44)	
	ADHD	16 (8.94)	
	Adjustment	10 (5.59)	
	Anxiety	37 (20.67)	
	Depression	9 (5.03)	
	Conduct ODD	12 (6.70)	
	CD	31 (17.32)	
	Other^†^	20 (11.17)	
	Psychosis	4 (2.23)	
	Somatic	7 (3.91)	

* Note: Though total sample was 320 referred and 601 non-referred sample, this table includes only dataset used for analysis, after exclusion for missing data.

† 'Other' category consists of conditions with only few children with specific learning disability, school refusal, stuttering, borderline traits, bipolar disorder.

In the initial runs of principal component analysis (PCA), eight components (factors) were identified, but 11 items with cross-loadings were removed as PCA components must be un-correlated (orthogonal). A second run with 76 items revealed two more crossloading items, leading to their exclusion. The final PCA, conducted on the remaining 65 items, retained six components supported by scree plot as well as Horn's parallel analysis, and this model explained 49.49% of the total item variance, with individual components contributions ranging from 3.76% to 18.90% (Table 2).

**Table 2:** Factor loadings and communalities based on a principal component analysis with varimax rotation for 65 items (N = 179)

Item no	Items (English translation)	1	2	3	4	5	6	Communality
18	Constantly moving (Can't stay quiet, restless, cannot stay at one place)	0.76						0.66
31	Disturbs others	0.74						0.63
1	Difficulty in completing any started work	0.72						0.61
11	Repeats mistakes	0.72						0.61
9	Changes his/her mood, distracted	0.71						0.60
20	Does not obey or do what is said	0.70						0.55
28	Scolds	0.70						0.65
21	Plays without caring	0.70						0.56
32	Fights	0.69						0.58
24	Keeps arguing and debating, and talks back	0.68		0.35				0.68
19	Stubborn	0.68						0.54
14	Cannot wait	0.67						0.55
25	Destroys things, vandalize	0.67						0.54
33	Teases	0.66						0.49
85	Breaks rules or tries to break	0.65						0.53
2	Cannot or does not pay attention	0.64				0.37		0.57
29	Hits or harms others	0.64					0.34	0.58
60	Speaks too much	0.62	0.32					0.52
58	Forgets (about studies, or other things)	0.59						0.50
4	Repetitive behaviors	0.58				0.32		0.50
23	Talks rudely with others	0.57		0.31				0.49
26	Runs away from home	0.57						0.43
22	Screams or shouts	0.57						0.42
56	Speaks lies	0.55						0.39
65	Behaving like kids	0.52					0.30	0.45
8	Doesn't want to do household works	0.51		0.36				0.40
83	Problem of overeating	0.44						0.30
57	Steals	0.43					0.30	0.36
87	Repeatedly thinking about making mistakes or remains cautious about hurting oneself/others		0.77					0.65
88	Repeatedly making sure if things are in okay or not, or right or wrong (Like: checking if the door is locked properly or not, whether the gas stove is turned off or not)		0.71					0.64
55	Keeps worrying for relatives		0.68	0.31				0.62
86	Worries about being dirty or about infections transmitted from germs, diseases		0.67					0.64
81	Complains of being haunted by any big worst incidents of life		0.64					0.54
76	Complains of remembering negative things that make him/her sad		0.62					0.57
61	Fears if someone scolds or complains		0.55					0.52
77	Taking drugs, drinking, and smoking		0.41					0.34
80	Worries about appearance of body		0.35					0.28
15	Feels hurt soon			0.63				0.48
6	Feels irritated and feels troubled			0.63				0.54
3	Many things going through his/her mind and worries a lot			0.63				0.55
7	Keeps crying			0.59				0.39
16	Takes same thing in mind for long time			0.59				0.44
74	Irritable in comparison to past			0.52				0.51
53	Does not feel to eat or have changed eating habits			0.47				0.29
5	Worries about own health			0.46				0.32
67	Seems upset			0.45		0.31		0.41
47	Restlessness				0.81			0.68
45	Complains of heart pounding				0.79			0.67
46	Breathlessness				0.77			0.71
44	Becomes unconscious, semi-conscious				0.63			0.52
59	Feels dizzy				0.60			0.43
49	Pain or Burning sensation at chest				0.60			0.44
48	Body pain				0.57			0.38
51	Headache			0.34	0.48			0.39
62	Startled		0.35		0.47			0.45
82	Tells about horrifying dreams or recurring dreams			0.34	0.40			0.39
79	Doesn't get enough sleep these days but still seems filled with energy.		0.34			0.55		0.46
36	Doesn't speak					0.55		0.36
38	Can't socialize or adapt					0.55		0.43
73	Not enjoying any activities (including games, work or in groups) in comparison to the first time					0.48		0.35
52	Insomnia					0.44		0.29
63	Speaks rough/swear words	0.32					0.58	0.46
78	Giving big talks or performs stunts putting life under risk in comparison to the past					0.34	0.55	0.54
70	Hearing voices that others do not.						0.54	0.48
13	Tells about dying or committing suicide						0.46	0.36

The six factors (components) were named based on their conceptual content and clinical relevance, with input from two mental health clinicians. The first factor, 'externalizing behavioral problems' includes items related to attention, hyperactivity, and conduct issues. 'Anxiety/Worries' factor encompasses items reflecting anxiety symptoms and worries. 'Upset/Sadness' reflects items associated with sadness, low mood, and emotional distress. 'Somatic concern' involves items related to physical complaints and bodily symptoms. 'Miscellaneous problems' comprises items related to sleep, socialization, and anhedonia, representing a mixed cluster of symptoms. Lastly, 'severe issues' includes items indicative of potentially severe psychopathology, such as hearing voices, suicidality, risk-taking, and aggression.

Regarding reliability, internal consistency coefficient was excellent for the 65 item version of the questionnaire and "Externalizing behavioral" factor (> 0.90) in both samples. For other factors it ranged from 0.83 to 0.62. Test-retest reliability was high for all factor scores (> 0.80) across one week based on 39 non-referred sample. Regarding cross informant correlation (parental agreement) among fathers and mothers, it was high for total score and all factor scores (0.74-0.85) based on 41 parents non-referred sample (Table 3).

**Table 3:** Reliability indices of 65-Item version

			Cronbach alpha	Test retest^†^	Cross-informant correlation^†^
ID	Factor/Scale	No of items	Referred (N=179)	Non-Referred (N=39)	Non-Referred (N=41)
EB	Externalizing/Behavioral	28	0.95	0.919^**^	0.850^**^
AW	Anxiety/Worries	9	0.85	0.891^**^	0.819^**^
US	Upset/Sadness	9	0.83	0.903^**^	0.742^**^
SC	Somatic concern	10	0.85	0.876^**^	0.846^**^
SM	Socialization/sleep/mood	5	0.62	0.870^**^	0.831^**^
SI	Severe issues	4	0.64	0.817^**^	0.738^**^
WS	65 item composite score	65	0.94	0.926^**^	0.921^**^

** Note: Indicates significance at p<.01 (two-tailed).

† Test-retest reliability and cross-informant agreement were assessed using Spearman's rank correlations in non-referred samples

The criterion validity evidence for the tool was assessed using multiple regression analysis, with referral status serving as the criterion/reference variable (Table 3). Referral status, age, sex, school type, grade, informant's relation with children and informant's education were included to find association with composite score of 65-item tool. Only referral status, age and informant's education were eligible for the final model based on simple linear regression results and multicollinearity analysis. The referral status emerged as a strong predictor (β = -6.958, p < 0.001) followed by age (β = 1.025, p < 0.001) while informant's education was not significant in the final model. Hence, the participants in referred group had high composite scores than the participants in the community whereas increasing age had more composite scores. Additionally, in the models for individual factors within the tool, referral status consistently demonstrated significant associations with Factor 1 (β = -2.538, p = 0.004), Factor 3 (β = -2.300, p < 0.001), Factor 4 (β = -3.603, p < 0.001), Factor 5 (β = -0.416, p = 0.001) and Factor 6 (β = -0.149, p < 0.001). Adjusted R-squared values ranged from 0.024 to 0.285 across the models, indicating that referral status, in combination with other predictor variables like age (for factor 2, 3, 4, 5, 6), sex (for factor 1, 3, 4), and school type (for factor 4), accounted for a meaningful proportion of variance in the tool's outcome measures.

Regression analyses were also conducted among referred group to assess whether clinical diagnosis predicted six factors scores. For the externalizing behavioural problems, clinical diagnoses including ADHD (β = 16.659, p < 0.001), conduct ODD (β = 7.034, p = 0.015), depression (β = -7.494, p = 0.022), and conversion dissociative (β = -4.168, p = 0.036) were significant predictors. For anxiety/worries factor, anxiety (β = 1.140, p = 0.049) independently predicted factor scores. For upset/sadness, diagnosis of depression independently predicted factor scores (β = 4.007, p = 0.001). For somatic concerns, conversion dissociative diagnosis independently predicted factor scores (β = 4.574, p < 0.001). For miscellaneous issues, ADHD (β = 0.851, p = 0.034), Somatic issues (β = -1.211, p = 0.035) and depression (β = 1.011, p = 0.049) significantly predicted this factor. Lastly, for severe issues, ADHD (β = 0.505, p = 0.018) and Psychosis (β = 2.067, p < 0.001) were independent predictors.

## DISCUSSION

This article summarises reliability and validity evidences for TEJ-CL as a parent report checklist for childhood psychopathology, which is intended as an add-on and symptom monitoring tool in clinical settings. The factor analysis yielded six interpretable factors, that capture a range of emotional and behavioral concerns, including externalizing behaviors, anxiety/worries, upset/sadness, somatic concerns, miscellaneous problems, and severe issues. These factors align conceptually with other established empirically based assessment tool i.e. the Child Behavioral Checklist (CBCL).^14^

Externalizing behavioral issues in this study emerged as first order factor, in contrast to this dimension being second order factor in CBCL. The items for attention problems are also merged with externalizing factor. It is possible that the Nepalese guardians have perceived attention issues, overactivity issues and academic issues as a cluster of 'under controlled' and externalizing issues. Anxiety/worries reflected various anxious symptoms, similar to anxiety scales in the CBCL. Upset/Sadness represented affective states distinct from anxiety, resembling the CBCL's Internalizing scale that includes depressive symptoms. Somatic Concern captured psychological distress through physical complaints, akin to the CBCL's Somatic Complaints scale. Miscellaneous Syndrome encompassed social and sleep-related issues, similar to features found in the CBCL's withdrawn/Depressed scale. Severe Issues comprised clinically significant problems, resonant with the CBCL's Thought Problems.

Reliability estimates using coefficient alpha were strong for first four syndromes which is comparable to established scales like CBCL and Conners' Rating Scale.^14,16^ This pattern aligns with previous reports on CBCL which reported higher reliability for total and externalizing/internalizing syndromes but lower reliability for thought problems across 31 countries using CBCL data.^17^ Test-retest reliability indices and cross-informant correlations among the parents were comparable to CBCL.^14^

In terms of criterion validity evidence, referral status was linked to elevated total composite scores and scores across individual factors, except for the anxiety/worries factor, which did not show a significant association. Notably, somatic symptoms demonstrated strong discriminatory power with referral status, aligning with prior research indicating that somatic complaints are prominent drivers of treatment referrals.^18,19^ Further criterion validity evidence was obtained for factor scores and diagnoses. The externalizing factor linked positively to ADHD, conduct disorder/Oppositional Defiant Disorder (ODD), and negatively to depression and conversion dissociative disorders. Similarly, the anxiety/worries factor aligned with anxiety diagnoses, while upset/sadness aligned with depression. The miscellaneous factor showed positive correlations with both ADHD and depression, but a negative correlation with somatic complaints. Finally, the severe issues factor correlated positively with psychosis and ADHD. This also implies that, the dimensional scores obtained from the tool can be utilized to assess categorical dimensions of psychopathology.^20,21^

The tool can assist clinician prior to, during or after regular clinical assessment as well as psychosocial intervention, as a comprehensive problem checklist which can assist in further assessment. The adult informants get the space to endorse the problem lists of their children. However, one should be cautious while using single composite score (e.g. total score) for few reasons. First, the problems items represent multidimensional construct as per factor analysis, and there is ongoing debate on interpretability of such total score as a single construct.^22^ Even as per current taxonomies in psychopathology, the construct of 'child psychopathology' or emotional behavioural problems are not unidimensional constructs. In addition, age range needs to be considered while interpreting total score as age and total score are positively associated in current study, which is also common trend in other studies.^23^

Regarding limitation, the overall research project did not integrate explanatory factors like stressors and biological determinants for emotional and behavioural problems. Similarly, the assessment strategy focussed solely on what was not 'going well,' ignoring the strengths of children for holistic assessment based approach.^24^ In addition, children's covert issues might have been ignored while solely basing on parental rating of checklist.^25^ Demographic and methodological limitations, such as non-representative sampling, not including ethnicity/cultural variation in design constrain the study's generalizability. Other issues include potential underestimation of clinically significant problems in the community sample which might range from 15-30%.^23^ Since, whole community sample was used as 'non referred,' and we did not screen for children at-risk, this would underestimate discriminating ability of the tool. TEJ-CL's ability to differentiate between individuals with and without emotional and behavioral problems using conditional probability estimates like sensitivity, specificity, positive predictive value, and negative predictive value has not been reported in this article, which would be relevant for screening in community will be published subsequently. Finally, further research on model fitting (e.g. confirmatory factor analysis) and large-scale community sample for development of norm is warranted.

## CONCLUSIONS

This study presents reliability and validity evidences of TEJ-CL in Nepalese context. The factor structure, reliability, and validity evidences are comparable to similar empirically and dimensionally based assessment tools in child psychopathology. Further research is needed to explore the generalizability and utility for community screening purpose.
